# Bull’s Head Sign in a Patient with SAPHO Syndrome

**DOI:** 10.4274/balkanmedj.galenos.2018.2018.1630

**Published:** 2019-02-28

**Authors:** Ufuk İlgen, Sezin Turan, Hakan Emmungil

**Affiliations:** 1 Department of Rheumatology, Trakya University School of Medicine, Edirne, Turkey

A 53-year-old male was evaluated for upper chest pain lasting 3 years. The pain was continuous, bilateral, progressive, and worse when the patient lay on his sides and during hyperabduction of the arms, radiating to the shoulders. Initially, it was responsive to non-steroidal anti-inflammatory drugs, but full daily doses of combined long- and short-acting non-steroidal anti-inflammatory drugs had failed in the last few months, resulting in impairment of daily activities and disturbance of sleep, causing the patient to seek medical help. On physical examination, the sternoclavicular, manubriosternal, and upper two costosternal joints on both sides were tender. Systemic examination did not reveal any additional abnormal findings. He had no other symptoms. A complete blood count, basic biochemical tests, the erythrocyte sedimentation rate, and serum C-reactive protein were normal. Serum levels of calcium, phosphorus, alkaline phosphatase, and plasma parathormone and 25-hydroxy vitamin D_3_ were normal as well. A posteroanterior chest radiograph did not reveal any significant findings. Bone scintigraphy with ^99m^Tc-methylene diphosphonate revealed increased osteoblastic activity in the manubriosternal, bilateral sternocostoclavicular, and lower cervical vertebral regions (Figure 1a). Computed tomographic examination of the anterior chest wall revealed hyperostosis and fusion of the first costosternal and manubriosternal joints (Figure 1b). ^18^F-fluorodeoxyglucose positron emission tomography and computed tomography fusion images showed increased activity in the sternoclavicular joints (arthritis) and hyperostotic lesions (osteitis) (Figure 1c). Written informed consent was obtained from the patient regarding the use of clinical information and imaging findings for educational and research purposes.

A diagnosis of Synovitis, Acne, Pustulosis, Hyperostosis, Osteitis syndrome was made, and treatment with intravenous zoledronic acid resulted in complete relief of pain. Due to its rarity, diagnosis of Synovitis, Acne, Pustulosis, Hyperostosis, Osteitis syndrome may be challenging in the absence of the full clinical picture, especially cutaneous manifestations (1,2). In this case, the diagnosis is guided mainly by typical radiological findings (3). Treatment options include non-steroidal anti-inflammatory drugs, corticosteroids, bisphosphonates, colchicine, and immunosuppressive or biological agents in refractory cases (1).

## Figures and Tables

**Figure 1:**
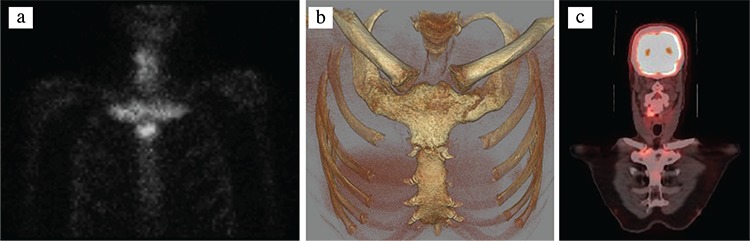
a-c. Anterior view of ^99m^Tc-methylene diphosphonate bone scintigraphy (a) showing increased osteoblastic activity in the manubriosternal, bilateral sternocostoclavicular (bull’s head sign), and lower cervical vertebral regions. Computed tomographic three-dimensional reconstruction of the bones of the anterior chest wall showing hyperostosis and fusion of the first costosternal and manubriosternal joints (b) and oblique coronal ^18^F-fluorodeoxyglucose positron emission tomography–computed tomography fusion image (c) showing increased activity in sternoclavicular joints (arthritis) and hyperostotic lesions (osteitis).
